# Experimental evolution of aging in a bacterium

**DOI:** 10.1186/1471-2148-7-126

**Published:** 2007-07-28

**Authors:** Martin Ackermann, Alexandra Schauerte, Stephen C Stearns, Urs Jenal

**Affiliations:** 1 Institute of Integrative Biology, ETH Zürich, CH-8092 Zürich, Switzerland; 2 Division of Molecular Microbiology, Biozentrum, University of Basel, CH-4056 Basel, Switzerland; 3 Department of Ecology and Evolutionary Biology, Yale University, New Haven, CT 06520-8106, USA

## Abstract

**Background:**

Aging refers to a decline in reproduction and survival with increasing age. According to evolutionary theory, aging evolves because selection late in life is weak and mutations exist whose deleterious effects manifest only late in life. Whether the assumptions behind this theory are fulfilled in all organisms, and whether all organisms age, has not been clear. We tested the generality of this theory by experimental evolution with *Caulobacter crescentus*, a bacterium whose asymmetric division allows mother and daughter to be distinguished.

**Results:**

We evolved three populations for 2000 generations in the laboratory under conditions where selection was strong early in life, but very weak later in life. All populations evolved faster growth rates, mostly by decreasing the age at first division. Evolutionary changes in aging were inconsistent. The predominant response was the unexpected evolution of slower aging, revealing the limits of theoretical predictions if mutations have unanticipated phenotypic effects. However, we also observed the spread of a mutation causing earlier aging of mothers whose negative effect was reset in the daughters.

**Conclusion:**

Our results confirm that late-acting deleterious mutations do occur in bacteria and that they can invade populations when selection late in life is weak. They suggest that very few organisms – perhaps none- can avoid the accumulation of such mutations over evolutionary time, and thus that aging is probably a fundamental property of all cellular organisms.

## Background

Aging seems paradoxical from an evolutionary perspective. Why is aging, which is detrimental for the individual, not eliminated by natural selection? Evolutionary theory provides an answer. Mutations that lead to aging are not efficiently removed by selection and can thus accumulate in populations over evolutionary time. Selection against these mutations is weak because under natural conditions, most individuals die for other, external reasons before aging manifests itself. This explanation hinges on two assumptions. First, such mutations must be detrimental late in life, but neutral [1] or beneficial [2] early on. If they were also detrimental early in life, they would be removed by selection. Second, the negative effects of age must be confined to old parents and to the progeny of old parents [3], while the progeny of young parents emerge rejuvenated. Rejuvenation refers to the fact that progeny are composed of newly synthesized organs, tissues, cells, and subcellular structures. As a consequence, they are less affected by the phenotypic deteriorations experienced by their aging parents. Without rejuvenation, negative effects would accumulate from generation to generation, and aging lineages would disappear [4].

Mutations with a negative effect that is specific for late age and can be rejuvenated in the progeny play a pivotal role in the evolution of aging. Any organism in which such mutations occur should evolve aging, whereas organisms in which such mutations do not occur should not age and should be potentially immortal. Initially, it was thought that such mutations can only occur in organisms with a distinction between soma and germline [4] where the negative effect of age would be confined to the soma and not be passed on to the progeny produced from the germline. Then, as anticipated by Weismann [5] the criterion for aging was expanded to any organism where the individuals emerging from reproduction are systematically different [6,7]. Aging has now been shown in unicellular eukaryotes [8,9] and even in bacteria [10,11]. As in other organisms, aging manifests in bacteria as decreasing performance with age. In bacteria, it is difficult to disentangle survival and reproduction, and it is thus not possible to use increasing mortality with age as an indicator for aging. Aging is thus quantified as a decline in the product of survival and reproduction [12] or as a decreasing growth rate with age [11].

According to the evolutionary explanation of aging, finding aging in bacteria suggests that mutations with deleterious effects specific to late life do occur in bacteria, and that they invaded populations over evolutionary times because selection late in life is weak. However, the existence of such mutations has yet to be demonstrated. That they do not necessarily occur is indicated by the failure to find them in viruses [13].

A second issue with such mutations is that they tie the evolution of aging to environmental conditions. One prediction is that fast aging should evolve if the strength of selection declines quickly with age, and slow aging if the strength of selection declines slowly. This prediction has been supported by a laboratory evolution experiment with fruit flies [14]. In this experiment, the decline in the strength of selection with age was varied by adjusting external mortality imposed by the experimenters. Another recent experimental study investigated the evolution of aging in natural populations of guppies living with or without predators [15]. The surprising outcome was that guppies in the presence of predators evolved slower aging. One plausible explanation for this result is that high extrinsic mortality does not always lead to weak selection late in life, because it may also cause a reduction in population density. If reduced population density benefits older individuals more than younger individuals, then increased extrinsic mortality might indeed improve the prospect of older individuals and thus lead to a slower decline in the strength of selection with age [16]. An alternative explanation is that if mutations with age-specific effects are rare, extrinsic risk does not easily modulate intrinsic aging.

Here, we used experimental evolution with bacteria to test the evolutionary theory of aging at a basic level of biological organization: populations of unicellular organisms. Initially clonal populations were allowed to evolve under conditions where selection late in life was very weak. We then tested whether those populations would evolve earlier aging. This experiment is a stringent test of both the assumptions and the predictions of the evolutionary theory of aging. It tests both the assumption that mutations with a negative effect that is specific for late age and is subject to rejuvenation do occur, and the prediction that such mutations can increase in frequency under conditions where selection late in life is weak. If the assumptions of this theory are met in these asymmetrically reproducing bacteria, one would expect them to hold in all cellular organisms where reproduction is not completely symmetrical.

## Results and Discussion

**Figure 1:**
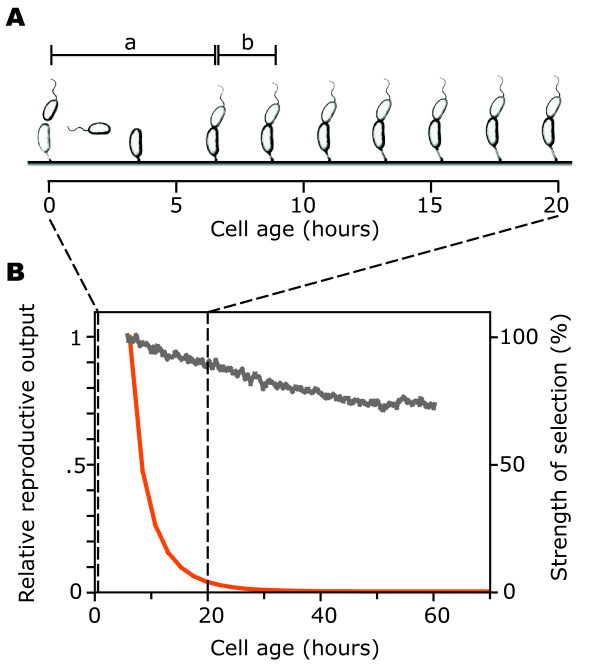
Life history of *C. crescentus *and selection imposed during experimental evolution. **(A) **The life cycle of *C. crescentus *begins with the release of a swarmer cell (at age 0). The swarmer cell differentiates to a stalked cell and divides for the first time at age *a*. The second division occurs at age *a *+ *b*. The interval between divisions, *b*, is nearly constant early in life. The time axis is drawn to scale for *C. crescentus *strain UJ590; **(B) **The reproductive output of stalked cells of strain UJ590 decreased with increasing age (grey line, left axis; average over nine independent experiments with 30 cells; data from [12]). During experimental evolution, selection was very strong early in life, and decreased with increasing age. The red line (right axis) denotes the reduction in fitness through a mutation that leads to death at a given age, as a function of age. Death before the onset of reproduction leads to a reduction of fitness by 100%.

We worked with *C. crescentus*, a bacterium where the two cells emerging from reproduction can be distinguished, and where one can be regarded as a mother and the other as a daughter. The life cycle of *C. crescentus *[17] begins with a motile swarmer cell (Fig. 1a). After dispersal the swarmer differentiates into a stalked cell, which can attach to a solid surface by means of a polar holdfast. It then initiates an asymmetric cell division that results in a motile swarmer progeny cell and in the sessile stalked mother cell. Stalked cells of *C. crescentus *age, manifested as a decrease in progeny production after many rounds of division [10,12](Fig. 1b).

To investigate the evolution of aging, we created conditions where the age-distribution was biased to young age, and most cells died before reaching advanced age. Populations of *C. crescentus *were cultured in liquid medium under conditions of exponential growth. Every 24 hours, small aliquots of cells were transferred to a fresh batch of medium, and the cells that were not transferred were discarded. The amount transferred was adjusted so that resources never became limiting and the populations grew continuously. Under this demographic regime, the population reached an age-distribution strongly biased to young age classes with about half of the cells being new-born, a quarter one division old, an eighth two divisions old, and so on. The populations contained very few cells that were several divisions old, because most cells were killed at transfer, and the populations were expanding exponentially between transfers.

We used a simple mathematical model from life history theory to calculate the strength of selection under these conditions as a function of an individual's age (see Methods). We calculated the fitness cost of hypothetical mutations that would lead to cell death at a given age, and plotted this cost as a function of the age at death. This analysis showed that selection was very strong early in life, and very weak late in life (Fig. 1b). Specifically, performance after an age of about 30 hours was virtually free of selection. Under these conditions, mutations with a deleterious effect specific to late age are close to neutral. Also, large negative effects at later age could be compensated for by a small advantage early in life. As an example, consider a mutation that would lead to cell death at an age of 48 hours. Under the conditions of our experiment, the fitness cost of this mutation is so small that it could be compensated for by a decrease in the age at first division of one second (see Methods). We thus expected mutations that improve early performance to sweep through the populations, leading to an increase in population growth rate reflected in a decrease in doubling time. If any of the mutations fixing in the experimental populations had costs at later age, we would expect to observe an evolutionary change towards faster aging.

### Evolutionary Changes

**Figure 2:**
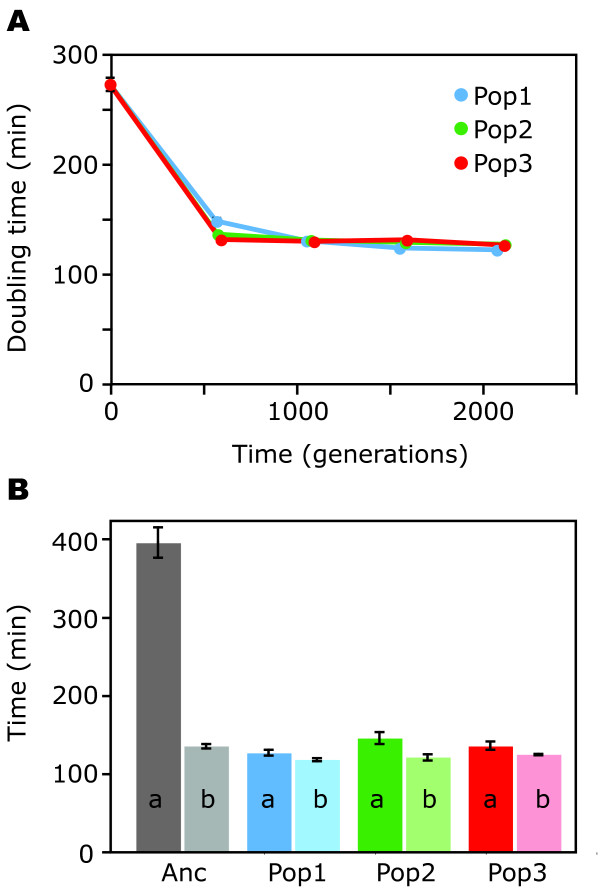
Life history changes during experimental evolution. **(A) **The population doubling time decreased by more than a factor of 2 in the course of 2000 generations of evolution. At each time point, five clonal isolates per population were analyzed. Error bars denoting standard error of the mean are smaller than the size of the symbols, except for time point 0; **(B) **Age at first division, *a*, and the average interval between consecutive divisions in the 20 hours after the first division, *b*, of ancestor and five isolates from each population after 1000 generations. All populations differed significantly from the ancestor in both traits (p < 0.05). The difference between ancestor and evolved populations is large in the age at first reproduction, and small in the interval between consecutive divisions. Abbreviations: Anc: ancestor; Pop1 to Pop3: population 1 to population 3.

We evolved three populations of *C. cresecentus *under these conditions. Every day, we determined the doubling time of every population (as described in the methods). At regular intervals, samples were taken from these populations and stored at -80°C. After 2000 generations, we revived aliquots from the frozen stocks as well as from the ancestor and measured population doubling times. These measurements showed that during 2000 generations of experimental evolution, population doubling time decreased by more than a factor of two (Fig. 2a). This finding was supported by an analysis of the daily measurement of population doubling time (Additional file 1). The daily measurements showed that population doubling time did not change substantially during the first 150 divisions, and then showed a rapid, marked decrease. This pattern is characteristic of the selective sweep of a beneficial mutation in asexual populations of microorganisms [18]. This and the fact that the changes in doubling time were stably inherited even after freezing and reviving strongly suggest that the decrease in population doubling time is a consequence of mutations that fixed in the experimental populations, and not simply a physiological adaptation to the new conditions by means of gene regulation.

Most of the reduction in population doubling time occurred during the first 1000 generations and resulted from a marked decrease in age at first division (Fig. 2b). A decrease in the age at first division is synonymous with a shortening of the period spent as a motile swarmer cell. In the natural environment, the motile swarmer cells disperse [17], and this might select for a long swarmer phase. In the homogenous environment used for experimental evolution, dispersal is no longer beneficial, and the swarmer phase thus presumably dispensable. The interval between two divisions of young stalked cells, in contrast, decreased only marginally (Fig. 2b). This result confirms that these conditions lead to the evolution of improved performance early in life and suggests that the length of the inter-division interval is constrained by cell cycle processes.

Next, we looked for changes late in life. We analyzed five clones from each population isolated after 2000 generations of evolution. For each clone, a cohort of individual stalked cells was followed for 60 hours with direct observation in a microscopy flow chamber [10], and division events were recorded. In this system survival and reproduction cannot be disentangled because the viability of cells can only be assessed when they reproduce. Thus, we cannot use measures of aging that rely on that distinction [19]. Instead, we used a measure for age-specific performance that combines these two fitness components: the reproductive function [20], i.e. the number of progeny produced per member of the cohort per hour, as a function of the age of the cohort. We measured the rate of aging as the rate at which the reproductive function decreased with age. To extract this quantity from the division record of individual cells, we used logistic regression to estimate the change with age in the probability that a member of the cohort produced a progeny per unit time.

**Figure 3:**
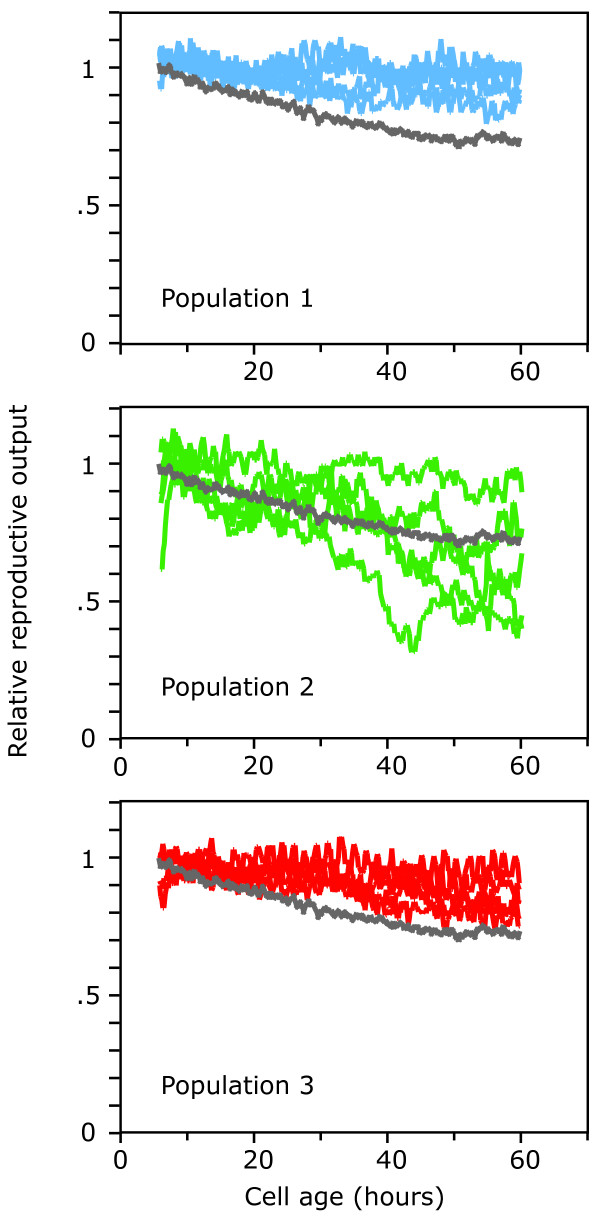
Changes in aging after 2000 generations of evolution. Population 1 (top panel) and population 3 (bottom panel) showed a slower decline in the reproductive function with age than the ancestor (grey line). The difference to the ancestor is significant (at p < 0.001) for both populations. Population 2 (middle panel) shows variation in the decline of the reproductive function with age. From each population, 30 or more cells from 5 independent clones were assayed, and each line represents the moving average of measurements from one clone, standardized to an intercept of 1. Statistic analysis was performed on the non-transformed data with logistic regression.

Contrary to expectation, populations 1 and 3 evolved slower aging than the ancestor, as indicated by a slower decrease in the reproductive function with age (Fig. 3). A slower decrease in the reproductive function means that stalked cells survived better to late age or reproduced at a higher rate late in life than the ancestor. In these populations, the number of progeny contributed by old stalked cells, which were almost completely free of selection, increased more than the number of progeny contributed by young stalked cells, which were under stronger selection (Fig. 3).

**Figure 4:**
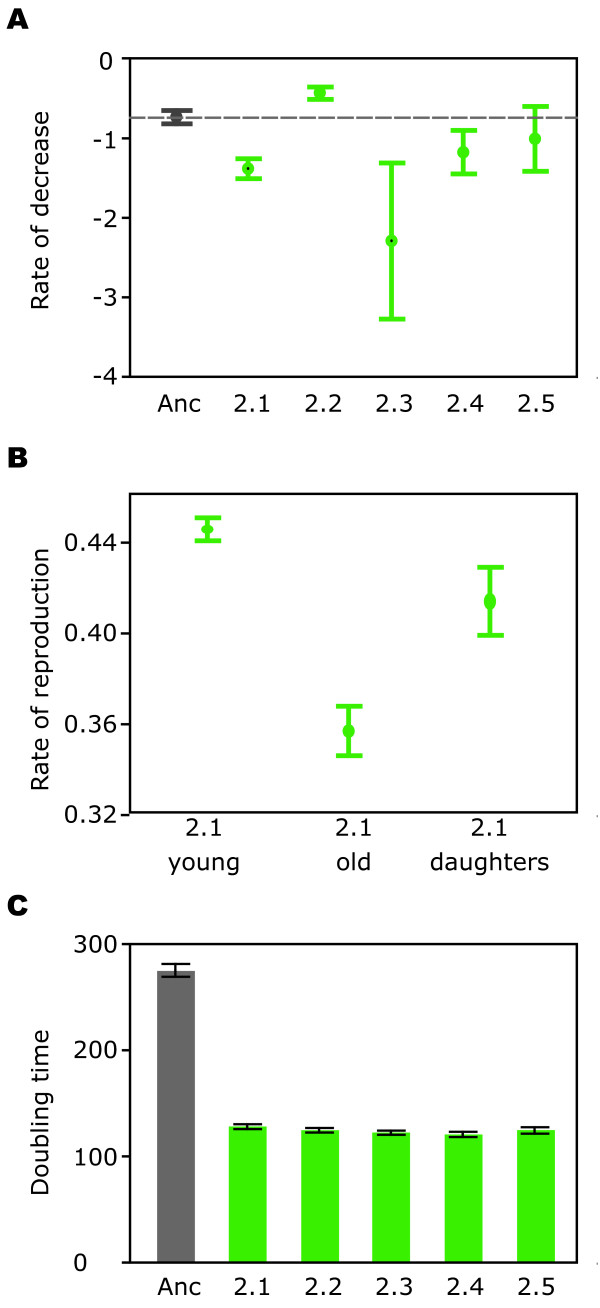
Faster aging and rejuvenating reproduction in population 2 after 2000 generations of evolution; **(A) **Rate of decrease in the reproductive function with age for the ancestor (grey symbol and hatched line) and five clonal isolates from population 2. The rate of decrease in the reproductive function with age (y-axis) was determined by logistic regression as described in the Methods. Isolate 2.2 declined slower and isolates 2.1, 2.3 and 2.4 declined faster than the ancestor (significant at p < 0.05. After correction for multiple testing, isolate 2.1 is significantly different from the ancestor); **(B) **Reproductive output (average number of progeny produced per hour over a period of twelve hours) of 204 pairs of mothers and daughters from isolate 2.1. While young mothers (2.1 young) reproduced quickly, their reproductive output at an average age of 18 hours (2.1 old) was lower. Daughters born to these mothers (2.1 daughters) reproduced quickly again. Old mothers were significantly different from young mothers and daughters (p < 0.001, after correction for multiple testing). Young mothers were not significantly different from daughters; **(C) **Population doubling time for the ancestor and the five isolates from population 2. The five isolates were not significantly different from one another. All error bars are standard error of the mean.

In population 2, the five clones isolated after 2000 generations showed striking variation in the rate of aging (Fig. 3). We repeated the measurements with each clone, and found that one clone showed slower aging and three showed faster aging than the ancestor (Fig. 4a). This result suggested that in population 2 at least one mutation that led to faster aging had become frequent, either because it conferred a benefit early in life and was thus selected for, or because it was neutral early in life and drifted into the population. The first scenario corresponds to the evolution of earlier aging by means of antagonistic pleiotropy [2], the second by means of mutation accumulation [1].

A mutation that leads to decreased performance in older cells can only reduce its negative impact if its deleterious effect is confined to a mother cell whose progeny is born rejuvenated. We tested for rejuvenating reproduction in one of the clonal isolates from population 2 that showed earlier aging. We recorded the reproductive output of stalked cells at the beginning of life and at an age of 18 hours, when they already showed a declining reproductive function. We then measured the reproductive output of daughters born to these aging mothers (this was possible because a mutation that fixed in this population led to an increased level of attachment in the microscopy chamber). The daughters had a higher reproductive output than their aging mothers, indicating that they were rejuvenated (Fig. 4b). There was a tendency for the reproductive output of the daughters to be lower than the reproductive output of their mothers measured early in life, but the difference was not statistically significant (a power analysis showed that a difference in reproductive output of at least 10% would have been recognized as significant with a probability of 80%). Such a fitness reduction in progeny born to old parents has been reported for organisms ranging from bacteria [11] to humans [21] and can be understood as consequence of weak selection on old parents [3]. As long as this effect only manifests in old parents and does not accumulate over generations, it does not lead to a successive deterioration of the aging lineage [3].

Because the negative effect of this mutation that led to earlier aging in population 2 was confined to late age, and performance at late age was inconsequential for fitness under these conditions, we expected that this mutation would not have an adverse effect on the growth rate. We measured the growth rate of the five isolates from population 2 and found no evidence for a fitness difference between the isolates with faster aging and the one isolate with slower aging (Fig. 4c). Importantly, these isolates all grew much faster than the ancestor, indicating that the isolates that showed faster aging were not generally defective. The fact that the growth rates were indistinguishable means that we cannot tell whether the mutant with faster aging was in the process of rising to fixation, or whether it was close to or at fixation and about to be replaced by another mutant with slower aging.

### Inference of the stage-specific risk encountered by the ancestors in nature

In the *Caulobacter *wild-type strain used as ancestor for the evolution experiment, the performance of stalked cells declines only slowly with age; some cells produced up to 130 divisions in 300 hours [10]. This indicates that the ancestor does not contain alleles leading to aging as early as observed in population 2. What prevents such alleles from invading natural populations of *C. crescentus*? To answer this question, one has to try to understand how selection acts in the natural environment of these bacteria.

The age-specific strength of selection in nature depends on extrinsic risks of mortality for juvenile swarmer and adult stalked cells. If extrinsic risk in the wild is exactly equal for swarmer and stalked cells, then most stalked cells would die before dividing many times. This is a consequence of the fact that, due to density regulation, natural populations do not grow without limit; in the long run, out of two cells that arise through division, one will die before dividing again (see also additional file 2). If swarmer and stalked cells were equally likely to die, then only about one in 10^30 ^stalked cells would reach an age of 100 divisions (2^100 ^≈ 10^30^). As the total number of bacteria (all species combined) on earth has been estimated to be around 10^30 ^[22], such great age would never be realized by *C. crescentus*. It would therefore be surprising that, when measured in the laboratory, stalked cells can still function at ages of 100 divisions and more *if *their mortality rate in the wild had been as high as that of swarmer cells.

One explanation for this puzzle is therefore that extrinsic risk in nature is much higher for juvenile swarmer cells than it is for adult stalked cells. Under those conditions, many cells would die during their swarmer phase; the few that would survive to become a stalked cell, however, would live a relatively safe life. Consequently, a substantial fraction of the cells that survive the swarmer phase would continue to survive for many divisions and reach a relatively high age. This would increase the importance of the stalked phase and would select for cells that are still able to reproduce even at an advanced age. This would explain why stalked cells of *C. crescentus *have such a long maximal lifespan [10].

Quantitative estimates of extrinsic risk in the natural environment of *C. crescentus *are not available, and it is thus not possible to quantify the scenario described above. However, observational data suggests that it might be qualitatively correct. Direct observation [23] and microcosm studies [24] indicated that stalked cells are resistant to protozoan grazing, while swarmers presumably are susceptible [23]. We used a simple mathematical model to investigate how differences in extrinsic risk between swarmers and stalked cells determine the age-specific strength of selection in the wild (additional file 2). This analysis confirms that if external risk is higher for swarmers than for stalked cells, the strength of selection decreases only slowly with age, and mutations with a deleterious effect that manifests after a few dozen divisions would be eliminated by selection. If this view is correct, it would mean that we shifted *C. crescentus *from the natural environment where stalked cells were relatively safe, and selection against early aging thus substantial, to an artificial environment where most stalked cells were killed early on, and selection against early aging was thus weak.

### The two main insights

Our results lead to two main insights. The first concerns the question, which organisms should age? Aging should evolve in any organism where mutations can negatively affect performance late in life without correlated costs early in life. Here, we show that a mutation with such an effect occurred in an experimental population of bacteria and rose to high frequencies under conditions where selection late in life was weak. While our results indicate that such mutations might be rare, the fact that they do occur in bacteria suggests that they might occur in all cellular organisms. This raises the question whether any cellular organism is immune to the accumulation of such mutations over evolutionary time, and thus to aging.

The second insight from this study pertains to how aging and lifespan evolve in response to external conditions. We tested the central prediction of the evolutionary theory of aging, that fast aging should evolve under conditions where selection late in life is weak. While we did find faster aging in one population, the dominant response was slower aging: the part of the life history under strong selection improved less than the part of the life history where selection was almost absent (Fig. 3). Similar discrepancies have been reported earlier [25,26]. Interestingly, here the reasons previously advanced to explain such discrepancies – that increased extrinsic mortality does not necessarily lead to weak selection late in life [16,27] – do not apply. The simplicity of our experimental system allows us to calculate the strength of selection as a function of age directly, and this analysis shows that during our evolution experiment the strength of selection indeed dropped very quickly with age (Fig. 1b).

The most likely explanation for our result is different, namely that mutations had unexpected phenotypic effects. We can draw two conclusions about the phenotypic effects of the mutations associated with the evolutionary changes in these populations. First, the mutations that increased growth rates by improving early life did not typically entail costs later in life. Second, some of the mutations that went to fixation had the correlated effect of leading to slower aging, even though selection late in life was very weak. These results echo conclusions drawn from experiments on fruit flies, which suggest that deleterious mutations with age-specific effects might be rare [28] and that in novel environments mutations can improve several fitness components simultaneously [29]. Our results go a step further. That the reproductive function shows a slower decrease with age in populations 1 and 3 than in the ancestor suggests the fixation of mutations that improved performance of stalked cells late in life even more than their performance early in life. It remains possible that continued selection in this novel environment might eventually reveal trade-offs between performance early in life and longevity, and lead to the evolution of faster aging in all populations.

## Conclusion

We conclude that mutations that contribute to aging can occur in bacteria, and that they can reach high frequencies if selection late in life is weak, as predicted by the evolutionary theory of aging. This suggests that this theory is generally valid for all cellular organisms. And we support previous findings that the evolution of aging can take unanticipated turns when mutations have unexpected phenotypic effects. Theory cannot reliably predict what kind of mutations should occur; it only predicts the fate of mutations with given effects. Understanding the evolution of aging requires experiments that provide information about mutational effects on aging. The experimental evolution of aging in a bacterium demonstrates that such model systems yield insights into fundamental aspects of aging.

## Methods

### Strains and growth media

The evolution experiment was initiated with *C. crescentus *UJ590 [10]. This strain derived from the wild-type strain CB15 ([17], ATCC 19089) by introduction of an in-frame deletion in the pilA gene (without this deletion, the aging assay in the microscopy flow chamber is not possible; presence of the adhesive pili prevents progeny cells from being removed from the flow chamber, so that they quickly accumulate). CB15 is a natural isolate that has been stored in the freezer and that is less adapted to the laboratory conditions than the common laboratory strains. The evolution experiment and all assays were done in M2G glucose minimal medium [30] supplemented with 25 mM CaCl2. PYE agar plates [30] were used where indicated.

### Evolution experiment

Populations were evolved in agitated cultures of 100 ml M2G at 30°C in Erlenmeyer flasks. In liquid culture, *C. crescentus *maintains its life cycle with the asymmetric cell division into a motile swarmer and a stalked cell, even though most cells do not attach, but float in the medium. Every 24 hours, the optical density (OD 660) was determined and the growth rate and population doubling time, *dt*, during the previous 24 hours calculated. Then, an aliquot of cells was transferred to a new batch of medium. The quantity of cells transferred was adjusted so that if the culture would continue to grow with the same doubling time, it should reach on OD 660 of 0.25 (about 2*10^10 ^cells) after another 24 hours of growth. The quantity to be transferred, *t *(in ml), was determined as

*t *= *OD*_goal _*100/(*OD*_current _* 2^24/*dt*^)

where *OD*_*goal *_is the goal optical density prior to the next transfer, chosen to be 0.25, and *OD*_*current *_is the optical density of the culture from which an aliquot was transferred. The factor 100 in the numerator accounts for the fact that the aliquot is transferred to 100 ml of medium. With this protocol, the population were always kept in exponential growth and never reached stationary phase. As population doubling time was measured every day, the decrease in population doubling time that occurred during the evolution experiment (Fig. 2a) could be compensated for by transferring smaller aliquots at the later stage of the experiment. The minimal population size that as transferred was about 10^6 ^cells.

A critical assumption of the evolution experiment was that the age-structure in the population was not altered at transfer. This required that a cell's chance of being transferred was independent of its age. This could be violated if a substantial fraction of cells would attach to the walls of the culture container. Such cells would then not have a chance of being transferred; if they were not representative of the population in terms of age, the age-structure in the population would be changed at transfer. To test whether this was a problem, we measured the number of cells that attached to glass under conditions of the selection experiment. We counted the number of cells attaching to glass slides that were immersed in the cultures during the 24 hour growth cycle, and extrapolated this number to the surface area of the flask. We estimated that less than 0.01% of all cells in the flask attached to the wall (fewer than two million cells were estimated to be attached to the walls at the time of transfer, while the total number of cells in the flask was about 2*10^10 ^cell). Even in the case that the cells that were attached were not representative for the population in terms of their age, their number is so small that the age distribution of the population would remain basically unaffected.

### Measuring growth rate and life history parameters

Phenotypic changes in the course of experimental evolution were measured on clonal isolates. To obtain clonal isolates, we streaked out aliquots of frozen samples (-80°) harvested from the evolving populations at regular intervals on PYE agar plates and isolated individual colonies. Growth rates (Fig. 2A) were measured in multiwell plates for five clonal isolates per population and time point, with three replicate cultures per clonal isolate. Comparisons of growth rates among clonal isolates from population 2 after 2000 generations (Fig. 4C) were based on 20 independent replicates per isolate. To measure the interval between two consecutive divisions early in life, *b *(Fig. 2B), five isolates from each population isolated after 1000 generations of evolution were analyzed. For each isolate, 30 stalked cells were observed from differentiation to an age of 20 hours in a microscopy flow chamber (see below). Cell division events were recorded, and *b *was defined as the average interval between two cell divisions in the 20 hours after the first division. The age at first division for each clonal isolate, *a *(Fig 2B), was determined by solving the Euler-Lotka equation numerically [20], based on the measured growth rate *r *and the interval between two consecutive divisions *b *early in life for that isolate. The relationship between the growth rate *r*, the age at first division, *a*, and the interval between divisions, *b*, is approximately defined as 1 = ∑_*n*=0 to ∞_e^-*r*(*a*+n*b)^. This is a simplified version of the Euler-Lotka equation for discrete time. Age-specific output is equal to one for all ages where a cell divides, and zero otherwise. This approximation assumes that cells continue to produce progeny at a constant rate throughout their life, thereby ignoring a decrease in the rate of progeny production and an increase in intrinsic mortality with age. However, these changes only manifest at later age, and performance late in life has only a very small effect in the Euler-Lotka equation because of the discounting factor e^-*r*(*a*+n*b)^. This simplified form of the Euler-Lotka equation gives therefore a very good approximation for the growth rate during the conditions of exponential growth encountered during the evolution experiment.

### Direct observation of individual cells and determination of the reproductive function

We used a method described in [10] to determine the reproductive output of individual stalked cells by means of direct observation in a microscopy flow chamber. In contrast to the procedure described in [10], cells were not synchronized prior to inoculation. Visual inspection suggested that the cells attaching during inoculation were swarmer cells, so that the experiment started with a cohort of cells of equal age. The physical condition in the microscopy flow chamber corresponded to the conditions during the evolution experiment; aerated M2G medium (30°C) flowed through the chamber, removing most of the progeny swarmer cells produced in the chamber, while the stalked cells remained attached. The number of progeny produced per individual of the cohort was determined every 10 minutes for 60 hours.

The number measured at a given age *x *corresponds to the product of the probability of surviving to that age, l(*x*), and the rate of reproduction at that age, m(*x*). This product is referred to as reproductive function k(*x*)[20]. In this experiment, it is not possible to determine whether cells that do not divide are dead or rather alive but not reproducing. As a consequence, it is not possible to divide the reproductive function k(*x*) into its two components, survival l(*x*) and reproduction m(*x*). However, how this division is done has no effect on the estimate of the age-specific contribution to progeny production, and we thus used changes in the reproductive function k(*x*) with age x as measure for the rate of aging. In the clonal isolates from population 2 after 2000 generations of evolution, a larger fraction of swarmer cells remained in the chamber (this population evolved increased adhesion). For these isolates it was thus possible to compare the reproductive output (defined as the progeny produced in a time interval) of pairs of mother and daughter cells. The reproductive output of 121 mothers was determined at the beginning of their life cycle and at an advanced age of on average 18 hours. These quantities were compared to the reproductive output of 204 daughter cells born to these mothers at an average of 18 hours. Reproductive output for young mothers, old mothers, and the daughters born to old mothers was measured during 12 hours, starting 4 hours after birth. This analysis is based on the assumption that the daughter cells that remain in the chamber are a representative sample of all daughter cells produced.

### Calculating the strength of selection as a function of age

To quantify the strength of selection, one first needs to identify the appropriate measure for fitness. In competition among clonally reproducing genotypes, the genotype with the highest population growth rate *r *will out-compete the rest [31] unless there are direct interactions between cells, for example through chemical inhibition. There is no indication that such direct interactions might play a role in this system, and we therefore used *r *as the measure for fitness. During experimental evolution, the effective long-term growth rate of the population was adjusted to zero by a density-dependent reduction in the population size during the daily transfer. However, as this reduction did not change the age-distribution of the population (see above), the evolutionary dynamics was determined by competition during the phases of exponential growth between transfers, and was unaffected by the population culling during transfers. The fitness sensitivity during exponential growth is thus the relevant measure for the strength of selection during experimental evolution.

We used the Euler-Lotka equation [20] to determine the fitness cost, in terms of a reduction in *r*, of a mutation leading to death after *m *divisions (Fig. 1B). Under the conditions during the evolution experiment, the fitness *r *of a strain is approximately defined by 1 = ∑_*n*=0 to *m*_e^-*r*(*a*+n*b)^, where *n *is a counter for divisions, *m *is the maximal number of divisions that a cell achieves, *a *is the age at first division, and *b *is the interval between consecutive divisions. As above, this is a simplified version of the Euler-Lotka equation for discrete time, where age-specific output is equal to one for all ages where a cell divides, and zero otherwise, and where a decrease in the rate of reproduction and an increase in intrinsic mortality with age are not included. Evaluating the equation numerically for different *m *reveals how *r *depends on *m*, and thus the fitness cost of a mutation that leads to death after *m *divisions (the fitness cost was calculated relative to a reference with *m *= 100. Using larger *m *as reference did not have an effect that was large enough to be detectable numerically). For plotting the age-specific strength of selection, we used chronological age rather than the number of divisions; in this approximation, the age at *n *divisions is *a*+n*b). To estimate the reduction in the age at first division required to compensate for death at a certain age *m*, we numerically determined the reduction in *a *(the age at first division) that was necessary to compensate for a fitness loss caused by death at age *m*.

### Statistical analysis

Statistical analysis was done with SPSS [32]. Changes in the reproductive function with age (Fig. 3 and Fig. 4A) were analyzed with logistic regression. The slope parameter beta of the logistic regression was used as a measure for changes in the division probability per unit time of a cell with age. Larger betas, indicating a faster decline in the reproductive function with age, were interpreted as faster aging. Each microscopy experiment yielded a separate measure of beta for the respective strain. The betas measured for different strains (Fig. 3 and Fig. 4A) were compared with ANOVAs. Fig. 4A is based on four independent experiments for isolates 2.1, 2.2 and 2.3, and three experiments for isolates 2.4 and 2.5. Where indicated, p-values were adjusted for multiple testing with Bonferroni-corrections in SPSS. For plotting (Fig. 1B and Fig. 3), the age-specific reproductive output was smoothed by calculating moving averages over 6 hours. Reproductive output of young mothers, old mothers, and daughters (Fig. 4B) was compared with ANOVA. For many mother cells, more than one daughter was analyzed. To account for the fact that daughters from the same mother were not statistically independent, we introduced a random factor in the analysis denoting the mother from which each daughter originated. Power analysis for the comparison between young mothers and daughters was done as described in [33] p. 164. For the power analysis, we calculated the average difference in the reproductive output between each mother and all of her daughters, and tested the sample of these differences for deviation from zero.

## Authors' contributions

MA formulated the question, planned the experiments, carried out the evolution experiment and the phenotypic assays, and wrote the manuscript. AS carried out phenotypic assays. SCS formulated the question and contributed to writing the manuscript. UJ formulated the question, planned the experiments and contributed to writing the manuscript.

## Supplementary Material

Additional file 1 Detailed description of the evolutionary changes in population doubling time. This file contains an additional figure (S1) depicting changes in the population doubling time during 211 days of experimental evolution, based on daily measurement, as well as a description of the methods. Click here for file

Additional file 2 The effect of juvenile mortality on the age-specific strength of selection. This file contains a mathematical model used for analyzing the effect of juvenile mortality on the age-specific strength of selection. The strength of selection was calculated for populations that grow exponentially (additional figure S2) and for populations that are at equilibrium (additional figure S3). Click here for file

## References

[B1] Medawar PB (1952). An Usolved Problem of Biology.

[B2] Williams GC (1957). Pleiotropy, Natural-Selection, and the Evolution of Senescence. Evolution.

[B3] Kern S, Ackermann M, Stearns SC, Kawecki TJ (2001). Decline in offspring viability as a manifestation of aging in Drosophila melanogaster. Evolution.

[B4] Rose MR (1991). Evolutionary Biology of Aging.

[B5] Weismann A (1882). Uber die Dauer des Lebens.

[B6] Partridge L, Barton NH (1993). Optimality, Mutation and the Evolution of Aging. Nature.

[B7] Jazwinski SM (1993). The Genetics of Aging in the Yeast Saccharomyces-Cerevisiae. Genetica.

[B8] Barker MG, Walmsley RM (1999). Replicative ageing in the fission yeast Schizosaccharomyces pombe. Yeast.

[B9] Mortimer RK, Johnston JR (1959). Life Span of Individual Yeast Cells. Nature.

[B10] Ackermann M, Stearns SC, Jenal U (2003). Senescence in a Bacterium with Asymmetric Division. Science.

[B11] Stewart EJ, Madden R, Paul G, Taddei F (2005). Aging and death in an organism that reproduces by morphologically symmetric division. PLoS Biology.

[B12] Ackermann M, Chao L, Bergstrom CT, Doebeli M (2007). On the Evolutionary Origin of Aging. Aging Cell.

[B13] De Paepe M, Taddei F (2006). Viruses' Life History: Towards a Mechanistic Basis of a Trade-Off between Survival and Reproduction among Phages. PLoS Biology.

[B14] Stearns SC, Ackermann M, Doebeli M, Kaiser M (2000). Experimental evolution of aging, growth, and reproduction in fruitflies. Proceedings of the National Academy of Sciences of the United States of America.

[B15] Reznick DN, Bryant MJ, Roff D, Ghalambor CK, Ghalambor DE (2004). Effect of extrinsic mortality on the evolution of senescence in guppies. Nature.

[B16] Abrams PA (1993). Does Increased Mortality Favor the Evolution of More Rapid Senescence. Evolution.

[B17] Poindexter JS (1964). Biological Properties + Classification of Caulobacter Group. Bacteriological Reviews.

[B18] Elena SF, Cooper VS, Lenski RE (1996). Punctuated evolution caused by selection of rare beneficial mutations. Science.

[B19] Partridge L, Barton NH (1996). On measuring the rate of ageing. Proceedings of the Royal Society of London Series B-Biological Sciences.

[B20] Charlesworth B (1994). Evolution in Age-structured Populations.

[B21] Gavrilov LA, Gavrilova NS, Robine JM, Kirkwood TBL, Allard M (2000). Human Longevity and Parental Age at Conception. Sex and Longevity: Sexuality, Gender, Reproduction, Parenthood.

[B22] Whitman WB, Coleman DC, Wiebe WJ (1998). Prokaryotes: The unseen majority. Proceedings of the National Academy of Sciences of the United States of America.

[B23] Poindexter JS, Pujara KP, Staley JT (2000). In situ reproductive rate of freshwater Caulobacter spp. Applied and Environmental Microbiology.

[B24] Jurgens K, Gude H (1994). The Potential Importance of Grazing-Resistant Bacteria in Planktonic Systems. Marine Ecology-Progress Series.

[B25] Gray DA, Cade WH (2000). Senescence in field crickets (Orthoptera; Gryllidae): examining the effects of sex and a sex-biased parasitoid. Canadian Journal of Zoology-Revue Canadienne De Zoologie.

[B26] Reznick D, Bryant M, Holmes D (2006). The evolution of senescence and post-reproductive lifespan in guppies (Poecilia reticulata). Plos Biology.

[B27] Williams PD, Day T (2003). Antagonistic pleiotropy, mortality source interactions, and the evolutionary theory of senescence. Evolution.

[B28] Pletcher SD, Houle D, Curtsinger JW (1998). Age-specific properties of spontaneous mutations affecting mortality in Drosophila melanogaster. Genetics.

[B29] Service PM, Rose MR (1985). Genetic Covariation among Life-History Components – the Effect of Novel Environments. Evolution.

[B30] Ely B (1991). Genetics of Caulobacter-Crescentus. Methods in Enzymology.

[B31] Charlesworth B (2000). Fisher, Medawar, Hamilton and the evolution of aging. Genetics.

[B32] SPSS (2005). SPSS.

[B33] Sokal RR, Rohlf FJ (1995). Biometry.

