# Supplementary material for: Experimental evolution of aging in a bacterium
Source: BMC Evol Biol. 2007 Jul 28;7:126. doi: 10.1186/1471-2148-7-126 (PMC2174458; doi:10.1186/1471-2148-7-126)
Supplement: Additional file 1 — Detailed description of the evolutionary changes in population doubling time. This file contains an additional figure (S1) depicting changes in the population doubling time during 211 days of experimental evolution, based on daily measurement, as well as a description of the methods. [file 1471-2148-7-126-S1.pdf]

Additional File 1: Detailed description of the evolutionary changes in population doubling time

Ackermann, Schauerte, Stearns and Jenal

In the course of the evolutionary experiment with *C. crescentus*, we determined the population doubling time of each of the three experimental populations on a daily basis (as described in the methods). The resulting data set can be used to describe how this quantity changed over time; Figure S1 depicts the time course of population doubling time during 211 days of experimental evolution. These 211 days correspond to the approximately 2000 generations of experimental evolution presented in Figure 1 (see main text). More precisely, during these 211 days, population 1 went through 2060 doubling, populations 2 through 2104 doublings, and population 3 through 2098 doublings.

The three populations show correlated fluctuations in the daily measurements of doubling time. These fluctuations presumably result from environmental influences – variation in the incubation temperature and in the quality of the growth medium. Despite these fluctuations, one trend is discernible: the population doubling time is approximately stable for an initial period of about 25 days (corresponding to about 160 generations). After 25 days, all three populations showed a decrease in doubling time. This pattern is characteristic for the selective sweep of a beneficial mutation in an asexual population, as for example described in (1).

Reference:

1. Elena, S.F., Cooper, V.S. and R.E. Lenski (1996) Punctuated Evolution Caused by Selection of Rare Beneficial Mutations. SCIENCE 272: 1802-1804.

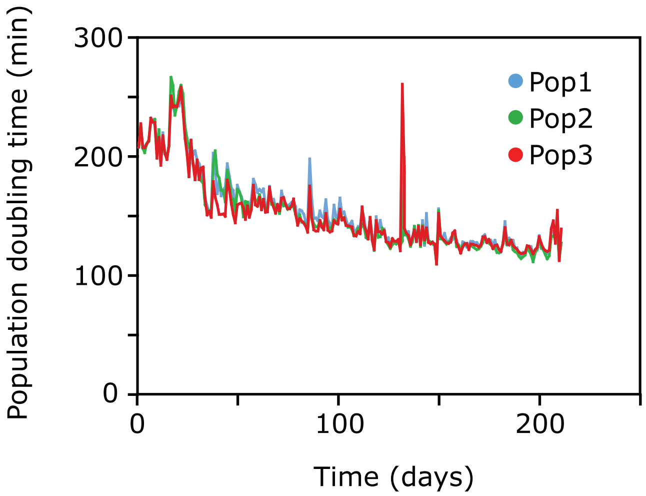

Figure S1: Changes in the population doubling time over 211 generations of experimental evolution, based on daily measurement. Abbreviations: Pop1 to Pop3: population 1 to population 3.
