# Supplementary material for: Experimental evolution of aging in a bacterium
Source: BMC Evol Biol. 2007 Jul 28;7:126. doi: 10.1186/1471-2148-7-126 (PMC2174458; doi:10.1186/1471-2148-7-126)
Supplement: Additional file 2 — The effect of juvenile mortality on the age-specific strength of selection. This file contains a mathematical model used for analyzing the effect of juvenile mortality on the age-specific strength of selection. The strength of selection was calculated for populations that grow exponentially (additional figure S2) and for populations that are at equilibrium (additional figure S3). [file 1471-2148-7-126-S2.pdf]

**Additional File 2: The effect of juvenile mortality on the age-specific strength of selection**

**Ackermann, Schauerte, Stearns and Jenal**

We used the Euler-Lotka equation [1] to determine how juvenile mortality would influence the age-specific strength of selection. As measure for the age-specific strength of selection we determined the fitness cost, in terms of a reduction in the population growth rate *r*, of a mutation leading to death after *m* divisions (as described in the main text). We then asked how the fitness costs changed if juvenile mortality was introduced. To implement juvenile mortality, we used a form of the Euler-Lotka equation that includes mortality: $1 = \sum_{n=0 \text{ to } m} e^{-r(a+n*b)} l_n$, where $l_n$ denotes to probability to survive to the $n^{th}$ division (the first division has the index $n = 0$). $l_0$ is the probability to survive to the first division, and 1- $l_0$ is therefore the juvenile mortality. We first assumed no adult mortality, so that $l_n = l_0$ for all n. Evaluating *r* as a function of *m* showed that with juvenile mortality the strength of selection remained high for longer than without juvenile mortality (Fig. S1). For example, if juvenile mortality is 0.9, a mutation that leads to cell death at an age of 30 hours decreases fitness by more than 50%; without juvenile mortality, the fitness cost of such a mutation would be virtually zero (Fig. S1; for plotting the figure we used chronological age rather than the number of divisions; the age at *n* divisions is $a+n*b$).

Without adult mortality (and with juvenile survival larger than zero), populations are not in equilibrium; they have a positive growth rate *r*, and the number of individual increases continuously. This is not realistic for natural populations, which have long-term growth rates that are very close to zero (otherwise they would increase in size without limits). It is thus interesting to investigate combinations of adult and juvenile mortality rates that lead to long-term growth rates $r = 0$. For $r = 0$, the Euler-Lotka equation reduces to $1 = \sum_{n=0 \text{ to } m} l_n$, where $l_n$ is again the probability to survive to the $n^{th}$ division. Writing *j* for the probability of surviving

the juvenile period ($j = l_0$), and $a$ for the (constant) probability of surviving between two divisions as a mother cell ($a = l_{n+1}/l_n$), the equation can be written as $1 = \sum_{n=0 \text{ to } m} j*a^n$ (because $l_0=j$, $l_1=j*a$, $l_2=j*a*a$, and so on). Re-arranging yields $1/j = \sum_{n=0 \text{ to } m} a^n$. For large m (m -> ∞) and $a$<1, the sum $\sum_{n=0 \text{ to } m} a^n$ approaches 1/(1-$a$), so that 1/$j$ = 1/(1-$a$), and $j$ = 1-$a$. This means that the long-term growth rate is zero if $j$+$a$ = 1, i.e., if the probability to survive the juvenile period and the probability to survive between two divisions as an adult sum up to one (and, likewise, the probability of dying during the juvenile period and the probability of dying between two divisions as an adult sum up to one). This simple result has an intuitive explanation: with $j$+$a$=1, at each cell division, on average one of the two cells produced survives to the next division. As a consequence, the number of cells stays constant over time. Combinations of juvenile and adult survivals that lead to growth rates of zero could result from density-dependent mortality. For example, if $a$ is constant (for example because it is determined by predation) and $j$ is regulated in a density-dependent manner, then $j$ will attain a value of (1-$a$) at equilibrium. There are currently no data available on survival rates of juvenile and adult *C. crescentus* in natural populations (such information could probably be obtained, because it is possible to determine the age, in numbers of divisions, of *C. crescentus* individuals collected from the wild [2]). We therefore analyzed different combinations of juvenile and adult survivals that lead to $r$ = 0, and calculated the fitness costs of mutations that lead to death at a given age (Fig. S2). These costs cannot be expressed as a percentage reduction of growth rate because the growth rate without mutations is zero. We thus calculated the costs in terms of the (negative) growth rate of a mutant in a resident population without this mutation. To do so, we solved the equation $1 = \sum_{n=0 \text{ to } m} e^{-r(a+n*b)} l_n$ for different $m$; the survival probabilities $l_n$ depended on $j$ and $a$ as described above. This analysis again shows that with high levels of juvenile mortality the strength of selection declines only slowly with age. For example, with juvenile mortality 1-$j$ = 0.95, a mutation leading to death after 50 divisions (corresponding to a chronological age of 116 hours) decreases the growth rate $r$ from zero to -0.002 [$h^{-1}$]. While it is not trivial to express this cost in terms of a fixation probability of this mutation, it seems substantial enough to prevent fixation of the mutation by

random genetic drift. In contrast, if juvenile mortality is low, the cost of such a mutation is much lower. For example, if 1-$j$ = 0.5, this mutation reduces the growth rate from zero to -$1*10^{-8}[h^{-1}]$; such a mutation is very close to neutral even in populations of substantial size.

**References:**

1. Charlesworth B (1994) Evolution in Age-structured Populations. Cambridge, UK: Cambridge University Press.

2. Poindexter JS, Pujara KP, Staley JT (2000) In situ reproductive rate of freshwater Caulobacter spp. Applied and Environmental Microbiology 66: 4105-4111.

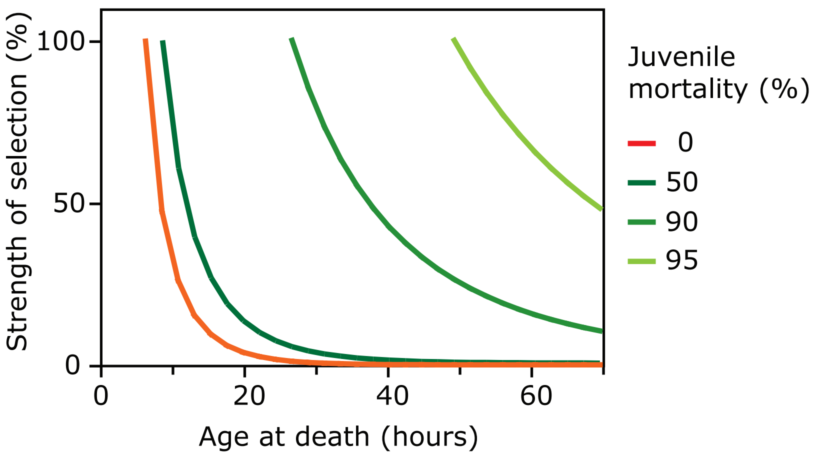


Figure S2: With juvenile mortality, the strength of selection declines more slowly with age than without juvenile mortality. The lines designate the reduction in fitness through a mutation that leads to death at a given age, as a function of age, for different levels of juvenile mortality. Juvenile mortality denotes the fraction of juveniles that die before the first reproduction. The red line represents the situation without juvenile mortality; this line is equal to the red line in Fig. 1. With increasing levels of juvenile mortality (green lines), selection remains substantial for longer. A fitness reduction of 100% (or higher) means that a mutation with such an effect would reduce the growth rate of the population to zero (or would lead to a negative growth rate). The calculations are based on measurement of the length of the juvenile phase and the interval between divisions made with the C. crescentus wildtype strain UJ590, as presented in Fig. 1.

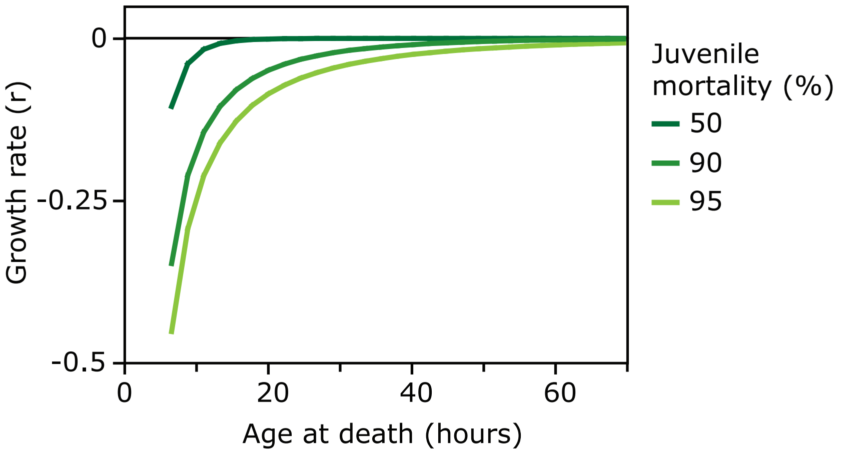


Figure S3: This graph illustrates the age-specific strength of selection in populations that have a long-term growth rate r = 0. In such populations, juvenile mortality and adult mortality sum up to one (see text). Starting with a cell type that does not have an upper limit for the length of the reproductive period (i.e., m = ∞) and that has r = 0, we determined the changes in growth rate induced by mutations that would lead to cell death at a given age. With low juvenile mortality (for example 0.5, meaning that juveniles and adults both have a chance of 0.5 to survive to the next division), the growth rate reduction introduced by such mutations becomes negligible very quickly for later age of onset. In contrast, if juvenile mortality is high (and, consequently, adult mortality low), mutations have a substantial fitness cost even if the age of onset is late. The calculations are again based on measurement of the length of the juvenile phase and the interval between divisions made with the C. crescentus wildtype strain UJ590, as presented in Fig. 1. The unit of r is h-1.
